# Astrocytic IP_3_Rs: Beyond IP_3_R2

**DOI:** 10.3389/fncel.2021.695817

**Published:** 2021-07-30

**Authors:** Mark W. Sherwood, Misa Arizono, Aude Panatier, Katsuhiko Mikoshiba, Stéphane H. R. Oliet

**Affiliations:** ^1^University of Bordeaux, INSERM, Neurocentre Magendie, U1215, Bordeaux, France; ^2^University of Bordeaux, CNRS, Interdisciplinary Institute for Neuroscience, IINS, UMR 5297, Bordeaux, France; ^3^ShanghaiTech University, Shanghai, China; ^4^Faculty of Science, Toho University, Funabashi, Japan; ^5^RIKEN CLST, Kobe, Japan

**Keywords:** astrocyte, inositol triphosphate (IP3) receptor, IP_3_R subtypes, calcium, GPCR, tripartite synapse, gliotransmission

## Abstract

Astrocytes are sensitive to ongoing neuronal/network activities and, accordingly, regulate neuronal functions (synaptic transmission, synaptic plasticity, behavior, etc.) by the context-dependent release of several gliotransmitters (e.g., glutamate, glycine, D-serine, ATP). To sense diverse input, astrocytes express a plethora of G-protein coupled receptors, which couple, via G_i/o_ and G_q_, to the intracellular Ca^2+^ release channel IP_3_-receptor (IP_3_R). Indeed, manipulating astrocytic IP_3_R-Ca^2+^ signaling is highly consequential at the network and behavioral level: Depleting IP_3_R subtype 2 (IP_3_R2) results in reduced GPCR-Ca^2+^ signaling and impaired synaptic plasticity; enhancing IP_3_R-Ca^2+^ signaling affects cognitive functions such as learning and memory, sleep, and mood. However, as a result of discrepancies in the literature, the role of GPCR-IP_3_R-Ca^2+^ signaling, especially under physiological conditions, remains inconclusive. One primary reason for this could be that IP_3_R2 has been used to represent all astrocytic IP_3_Rs, including IP_3_R1 and IP_3_R3. Indeed, IP_3_R1 and IP_3_R3 are unique Ca^2+^ channels in their own right; they have unique biophysical properties, often display distinct distribution, and are differentially regulated. As a result, they mediate different physiological roles to IP_3_R2. Thus, these additional channels promise to enrich the diversity of spatiotemporal Ca^2+^ dynamics and provide unique opportunities for integrating neuronal input and modulating astrocyte–neuron communication. The current review weighs evidence supporting the existence of multiple astrocytic-IP_3_R isoforms, summarizes distinct sub-type specific properties that shape spatiotemporal Ca^2+^ dynamics. We also discuss existing experimental tools and future refinements to better recapitulate the endogenous activities of each IP_3_R isoform.

## Introduction

Over the last three decades, Ca^2+^ imaging has revealed new roles for astrocytes. Indeed, astrocytic Ca^2+^ signaling was shown to regulate synaptic transmission, synaptic plasticity, and to influence behavior (Araque et al., 2014; Park and Lee, 2020). Inositol 1,4,5-trisphosphate receptors (IP_3_Rs) mediated Ca^2+^ signaling (IP_3_R-Ca^2+^) is regarded a primary generator of astrocytic Ca^2+^ signaling. Upon activation of G_q_-GPCRs, the main input pathway of astrocytes, phospholipase C breaks down PIP_2_ into DAG and IP_3_, activating IP_3_R predominantly located on the membrane of endoplasmic reticulum (ER) resulting in Ca^2+^ release (Bootman et al., 2001). This IP_3_R-mediated Ca^2+^ signaling is considered to trigger the activity-dependent and selective release of chemical transmitters (gliotransmitters) such as glutamate, D-serine, and ATP, which have distinct influences over neuronal activity. Initially, IP_3_R subtype 2 (IP_3_R2) was the only recognized Ca^2+^ channel in astrocytes; However, advanced Ca^2+^ imaging techniques have since identified novel Ca^2+^ sources, including mitochondria (Agarwal et al., 2017), transient receptor potential ankyrin 1 (Shigetomi et al., 2012, 2013b), L-type voltage gated Ca^2+^ channels (Letellier et al., 2016), sodium/calcium exchanger (Kirischuk et al., 1997; Boddum et al., 2016; Rose et al., 2020), and transient receptor potential canonical (Shiratori-Hayashi et al., 2020) amongst others, thereby expanding the known Ca^2+^ signaling toolkit of astrocytes. Doubtlessly additional Ca^2+^ channels and sources will emerge in the future.

While the field’s focus has moved on from understanding IP_3_Rs to identifying new Ca^2+^ sources, understanding IP_3_R signaling in astrocytes remains highly relevant. Indeed, IP_3_Rs are the primary target for manipulating astrocytic activity, and such manipulations have proven to be very consequential in many studies. Since most of these manipulations indiscriminately influence all IP_3_R subtypes, this could reflect the key role played by IP_3_R subtypes other than IP_3_R2, namely IP_3_R1 and IP_3_R3, which were mostly overlooked. In this review, we summarize the evidence for different subtypes of IP_3_R and discuss how we can better study the role of IP_3_R-Ca^2+^ signaling in astrocytes which is one of the core issues in understanding astrocyte physiology.

## Evidence for Three Subtypes

### The Dogma: IP_3_R2 the Sole Functional Astrocytic IP_3_R

There are three mammalian IP_3_R subtypes, i.e., IP_3_R1 (Furuichi et al., 1989; Mignery et al., 1989; Yamada et al., 1994), IP_3_R2 (Mignery et al., 1990; Südhof et al., 1991; Yamamoto-Hino et al., 1994; Iwai et al., 2005), and IP_3_R3 (Blondel et al., 1993; Yamamoto-Hino et al., 1994; Iwai et al., 2005). Among them, IP_3_R2 has widely been accepted as the only functional IP_3_R subtype present in astrocytes, and consequently, the IP_3_R2KO model mouse has been at the center of numerous important studies (Agulhon et al., 2010; Takata et al., 2011; Chen et al., 2012; Navarrete et al., 2012, 2019; Cao et al., 2013; Perez-Alvarez et al., 2014; Petravicz et al., 2014; Gómez-Gonzalo et al., 2015, 2017; Mariotti et al., 2016; Monai et al., 2016; Perea et al., 2016; Martin-Fernandez et al., 2017; Tanaka et al., 2017). Although an important model in the astrocyte field, the belief that knocking out IP_3_R type-2 abolishes IP_3_ induced Ca^2+^ release (IICR) entirely appears to need remedying. This section considers the historical data from which the dogmatic view of astrocytic-IP_3_R2 has flowed and reviews the evidence for other IP_3_R subtypes.

### Astrocyte Proteome

Several studies explored IP_3_Rs using immunohistochemistry which provided a consensus over the expression of IP_3_R2 in hippocampal/cortical astrocytes and Bergmann glia (Sharp et al., 1999; Holtzclaw et al., 2002; Hertle and Yeckel, 2007; Takata et al., 2011; Chen et al., 2012). While there are some conflicting reports over the immunoreactivity of IP_3_R3 in astrocytes and Bergmann glia (Sugiyama et al., 1994; Yamamoto-Hino et al., 1995; Hamada et al., 1999; Sharp et al., 1999; Holtzclaw et al., 2002), IP_3_R1 immunoreactivity was not initially observed in glia (Nakanishi et al., 1991; Dent et al., 1996; Hamada et al., 1999; Sharp et al., 1999; Holtzclaw et al., 2002; Hertle and Yeckel, 2007). These findings supported the view that IP_3_R2 is the predominant astrocytic IP_3_R. However, these results may also reflect limitations of the available IP_3_R antibodies or the difficulty of accurately assigning proteins located within ultrathin astrocyte processes, which are below the resolution limit of conventional microscopy (Panatier et al., 2014; Arizono et al., 2020) and buried amongst neuronal dendrites.

**FIGURE 1:**
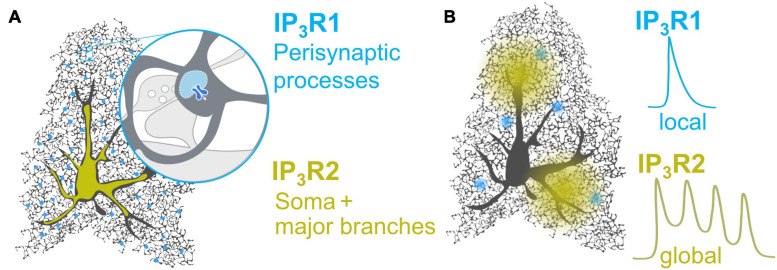
Proposed distribution and Ca^2+^ profiles of IP_3_R1 and IP_3_R2 in astrocytes. **(A)** While IP_3_R2 is distributed in the soma and major branches (Takata et al., 2011; Chen et al., 2012; Petravicz et al., 2014), IP_3_R1 may be enriched in the peri-synaptic processes (Sherwood et al., 2017; Takano et al., 2020), for instance in the nodes which were recently shown to host 2-APB-sensitive local Ca^2+^ signals at the tripartite synapse (Arizono et al., 2020). **(B)** IP_3_R2 generates global Ca^2+^ waves and oscillations in both astrocytes (Srinivasan et al., 2015; Sherwood et al., 2017) and model cells (Miyakawa, 1999). IP_3_R1 mediated Ca^2+^ dynamics in astrocytes have not been observed directly, but may resemble IP_3_R1-dependent monophasic Ca^2+^ transients observed in model cells (Miyakawa, 1999) and dendritic spines of pyramidal neurons (Holbro et al., 2009).

IP_3_R1 immunoreactivity was recently detected in spinal dorsal horn astrocytes (Shiratori-Hayashi et al., 2020) and, albeit with low stringency, in isolated astrocytes from adult mice (Chai et al., 2017). Using a state of the art TurboID construct to biotinylate proteins in the immediate proximity of tripartite synapses, Takano et al. (2020) report enrichment of IP_3_R1 protein in the peri-synaptic astrocytic compartment (Takano et al., 2020). This finding, however, should be interpreted with some caution as identified proteins were assigned to astrocytes based on published mRNA datasets (Zhang et al., 2014, 2016). Nevertheless, IP_3_R1 protein enrichment in fine astrocytic processes is consistent with Ca^2+^ imaging studies (Sherwood et al., 2017) and could account for the poor detection in various assays which favor detection in large subcellular compartments, i.e., major processes and soma of astrocytes. Notably, IP_3_R2, which is reported to be in the soma and main branches (Chen et al., 2012), was not enriched in peri-synaptic astrocytic compartments (Takano et al., 2020), likely reflecting the different subcellular distribution of IP_3_R1 and IP_3_R2 (Figure 1A and Table 1).

**TABLE 1:**
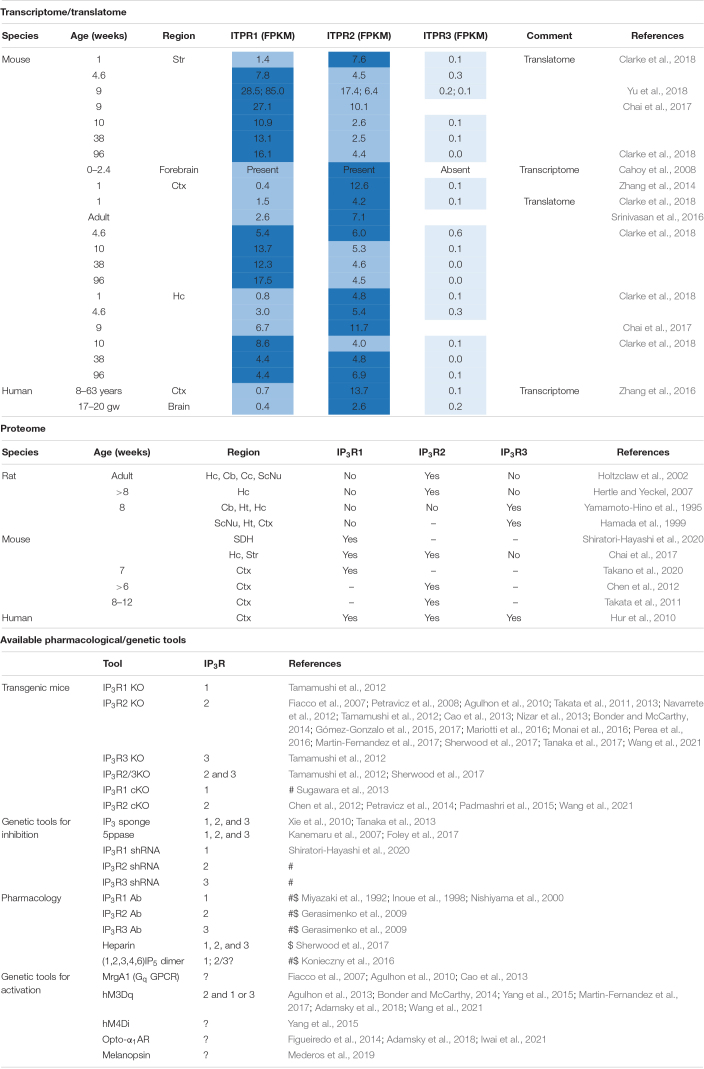
Dissecting IP_3_R subtypes.

*# Untested in astrocytes; $ requires loading using an astrocytic patch-pipette; ?, unknown; FPKM, fragments per kilobase of transcript per million mapped reads; Ctx, cortex; Str, striatum; Hc, hippocampus; Ht, hypothalamus; Cb, cerebellum; Cc, corpus callosum; ScNu, suprachiasmatic nucleus; SDH, spinal dorsal horn; gw, gestational weeks. Darker shades of blue represents higher FPKM values.*

### Astrocyte Transcriptome

IP_3_R1, IP_3_R2, and IP_3_R3 are encoded by the respective genes ITPR1, ITPR2, and ITPR3. Notably, mRNA for all three genes have been detected in astrocytes isolated from young and aged mouse brain (Cahoy et al., 2008; Zhang et al., 2014; Chai et al., 2017; Clarke et al., 2018) as well as humans (Zhang et al., 2016). Furthermore, ITPR1 and ITPR2 are actively translated in astrocytes of adult mice (Srinivasan et al., 2016; Chai et al., 2017; Clarke et al., 2018; Yu et al., 2018) indicating that their proteins are produced in astrocytes. In astrocytes the isoform transcript abundance is generally ITPR2 > ITPR1 >>> ITPR3 (ITPR3 mRNA is present in very small amounts and may be negligible). However, these transcripts are developmentally and differentially regulated across brain regions and in some instances ITPR1 mRNA appears to be more abundant than ITPR2 mRNA (Yu et al., 2018; Table 1).

### Ca^2+^ Imaging

Over the last decade, studies show that deletion of IP_3_R2 does not abolish Ca^2+^ signaling in astrocytes. It is now generally agreed that bulk/somatic cytosolic Ca^2+^ responses are hard to detect in IP_3_R2KO astrocytes (Petravicz et al., 2008, 2014; Agulhon et al., 2010, 2013; Takata et al., 2011, 2013; Nizar et al., 2013). However, with a detailed examination, rich Ca^2+^ activity can be observed within fine astrocyte processes (Di Castro et al., 2011; Haustein et al., 2014; Kanemaru et al., 2014; Srinivasan et al., 2015; Rungta et al., 2016; Agarwal et al., 2017; Sherwood et al., 2017). Non-IP_3_R2 Ca^2+^ activity has largely been interpreted as evidence for non-IP_3_R Ca^2+^ stores, nevertheless, these activities could equally arise from IP_3_R1 and or IP_3_R3 (Tamamushi et al., 2012; Sherwood et al., 2017). Indeed, Ca^2+^ release from the ER was recently reported for IP_3_R2KO astrocytes (Okubo et al., 2019). In Bergmann glia, while IP_3_R2 was shown to be a dominant subtype, IP_3_R1 and IP_3_R3 were also shown to contribute to rapid Ca^2+^ events in the processes (Tamamushi et al., 2012). Similarly, using IP_3_R2KO and IP_3_R2/3 double KO mice, we found that IP_3_R2 is involved in global Ca^2+^ release, whereas local Ca^2+^ signals involve IP_3_R1 and IP_3_R3 (Figure 1B) (Sherwood et al., 2017) in hippocampal astrocytes. Knock-down of IP_3_R1 in dorsal spinal cord astrocytes of IP_3_R2KO mice also unmasked IP_3_R1 mediated Ca^2+^ signals (Shiratori-Hayashi et al., 2020). Furthermore, the selective activation of G_q_-GPCR/IP_3_R/Ca^2+^ signaling in astrocytes, using DREADDs (designer receptor exclusively activated by designer drug, see section “Activation of IP_3_ Induced Ca^2+^ Release in Astrocytes”), triggered Ca^2+^ events in the astrocytic processes of IP_3_R2KO mice (Wang et al., 2021) indicating the presence of IP_3_R1/3.

2-APB was introduced as an antagonist of IP_3_Rs (Maruyama et al., 1997) and has been widely used to investigate the contribution of IP_3_Rs to cellular Ca^2+^ signaling. 2-APB appears to preferentially block IP_3_R1 and IP_3_R3, whereas cells predominantly expressing IP_3_R2 seem largely insensitive (Kukkonen et al., 2001; Bootman et al., 2002; Saleem et al., 2014). Thus, the fact that 2-APB reduces astrocyte Ca^2+^ amplitude and responsiveness in diverse brain regions (Sul et al., 2004; Young et al., 2010; Tamura et al., 2014; Tang et al., 2015; Arizono et al., 2020) and spinal dorsal horn (Shiratori-Hayashi et al., 2020) may support the presence of functional IP_3_R1 and IP_3_R3 in astrocytes. The functional role of non-IP_3_R2 in astrocytic Ca^2+^ signaling remains to be determined, it is possible that some membrane receptors are functionally coupled solely to IP_3_R1 or IP_3_R3 and generate local Ca^2+^ events, which may or may not be involved in triggering IP_3_R2 dependent Ca^2+^ waves (Petravicz et al., 2008). Alternatively, it is possible that IP_3_Rs exist as heterotetramers (Wojcikiewicz and He, 1995; Nucifora et al., 1996).

### Phenotypic Comparison

Comparison of WT and total IP_3_R2KO mice has revealed some important physiological roles of IP_3_R2 signaling in astrocytes, i.e., motor learning (Padmashri et al., 2015) modulating depressive-like behaviors (Cao et al., 2013) but this remains controversial (Petravicz et al., 2014). The possible roles of astrocytic IP_3_R1 and IP_3_R3 may be gleaned by comparing WT and IP_3_R2KOs with manipulations that impair all IP_3_R subtypes. For example, while IP_3_R2KO has no impact on sleep (Cao et al., 2013) overexpressing a transgene for the IP_3_ hydrolyzing enzyme, IP_3_-5-phosphatase, in astrocytes, suppresses Ca^2+^ release from all IP_3_R subtypes and disrupts sleep (Foley et al., 2017). Similarly, although IP_3_R2KO had no impact on hippocampal LTP (Agulhon et al., 2010), we showed that loading a single astrocyte with the membrane-impermeable pan-IP_3_R antagonist, heparin, blocks long-term synaptic potentiation (Sherwood et al., 2017). These studies, taken together, indicate that non-IP_3_R2 IP_3_Rs regulate sleep and hippocampal LTP.

### Summary

The expression of multiple IP_3_R subtypes in astrocytes has a wide-reaching implication in the field. Studies using IP_3_R2KO as a model for blocked IICR would need to be re-assessed to include the possibility of IICR mediated by IP_3_R1 and IP_3_R3.

## Prospectus/Advantage of Multiple Ip_3_R Isoforms

### Different Properties of IP_3_R Subtypes

The three IP_3_R subtypes share only 65–85% homology accounting for many of the subtype-specific properties leading to particular spatiotemporal features of Ca^2+^ responses. Although high homology is observed in regions critical for forming the IP_3_−gated Ca^2+^ channel, each subtype has a different IP_3_ affinity; IP_3_R2 > IP_3_R1 > IP_3_R3 (Zhang et al., 2011). High IP_3_ affinity of IP_3_R2 has been associated with slower kinetics and more prolonged duration of IP_3_R2-mediated Ca^2+^ microdomains, or Ca^2+^ puffs (Mataragka and Taylor, 2018).

Importantly, IP_3_R channel activity is not only regulated by IP_3_ but also by Ca^2+^ (Finch et al., 1991). The synergy between IP_3_ and Ca^2+^ creates repetitive IP_3_R activation and inhibition, resulting in Ca^2+^ oscillations. Such oscillations are crucial to protect cells from extended Ca^2+^ elevation that can often be toxic to the cell (Berridge et al., 2000). The oscillatory pattern is considered necessary for many cellular processes, such as fertilization (Miyazaki et al., 1992). IP_3_R2 mediates long-lasting, regular Ca^2+^ oscillations, whereas IP_3_R1 or IP_3_R3 tend to exhibit mono-phasic transients or very rapidly dampened Ca^2+^ oscillations (Miyakawa, 1999).

The difference in IP_3_R subtype properties is further characterized by various binding partners, including kinase and phosphatases, which can further fine-tune Ca^2+^ profiles. Interestingly, while there are many interacting partners common to all three IP_3_R subtypes, the nature of their regulation can be subtype-specific. For instance, protein kinase C, depending on the IP_3_R subtype, can either be stimulatory or inhibitory; this difference likely reflects isoform-specific phosphorylation sites. Further detailed biochemical study (Hamada et al., 2017) is required to investigate how various IP_3_R binding molecules specifically regulate each IP_3_R isoform (Hamada and Mikoshiba, 2020).

### Different Distribution and Role of IP_3_R Subtypes Within Various Tissues

One important feature that defines the subtype-specific role of IP_3_Rs *in vivo* is the tissue distribution patterns. While IP_3_R1 is mostly expressed in the central nervous system, IP_3_R2 and IP_3_R3 are broadly expressed in various organs such as the heart, pancreas, liver, and salivary glands (Hisatsune and Mikoshiba, 2017). This distribution pattern is tightly linked to the physiological role of IP_3_R subtypes. Reflecting its rich expression in Purkinje cells, mice lacking IP_3_R1 exhibit severe cerebellar ataxia, a seizure−like posture, impaired cerebellar LTD. (Inoue et al., 1998), and die within 3–4 weeks of birth (Matsumoto et al., 1996). The dysregulation of IP_3_R1 is linked with other brain disorders such as Huntington’s disease and Alzheimer’s disease (Hisatsune and Mikoshiba, 2017). IP_3_R2 is associated with sweating (Klar et al., 2014), bone formation (Kuroda et al., 2008), and heart hypertrophy (Nakayama et al., 2010; Drawnel et al., 2012; Vervloessem et al., 2015). IP_3_R3 plays a role in taste sensing (Hisatsune et al., 2007) and hair cycle (Sato-Miyaoka et al., 2012). IP_3_R2 and IP_3_R3 together are associated with the heart’s development (Uchida et al., 2010, 2016) and secretion of saliva and tears (Futatsugi, 2005; Inaba et al., 2014).

### Different Distribution and Role of IP_3_R Subtypes Within a Cell

Some cells express multiple IP_3_R subtypes, enabling each subtype to uniquely contribute to Ca^2+^ profiles and cellular functions. For instance, in HeLa cells, knock-down of IP_3_R1 terminates Ca^2+^ oscillations, whereas knock-down of IP_3_R3 results in more robust and long-lasting Ca^2+^ oscillations (Hattori et al., 2004). The specific contribution of IP_3_R subtypes can also depend on their distinct subcellular distribution, as seen in pancreatic acinar cells (Lur et al., 2011), COS cells (Pantazaka and Taylor, 2011), and DT40 cells (Bartok et al., 2019). In Bergmann glia, knocking out IP_3_R2 resulted in decreased agonist-induced Ca^2+^ release while knocking out IP_3_R1 and IP_3_R3 resulted in delaying the peak of agonist-induced Ca^2+^ release specifically in astrocytic processes (Tamamushi et al., 2012), suggesting their subtype-specific distribution. It is likely that astrocytes, which express multiple IP_3_R subtypes, also take advantage of subtype-specific distribution.

In astrocytes, IP_3_Rs are predominantly located on thapsigargin sensitive ER Ca^2+^ store. The ER in astrocytes may be found throughout the cell in the soma, major processes (Okubo et al., 2019, 2020), and peri-synaptic astrocytic processes (Bergersen et al., 2012), in close association with adhesion junctions (puncta adherentia) between dendritic spines and astrocytic processes (Spacek and Harris, 1998). Although recent studies indicate that ER and other membrane-bound organelles are absent from peri-synaptic processes (Patrushev et al., 2013) this likely reflects their sensitivity to chemical fixation (Korogod et al., 2015). The ER store in astrocytes is heterogeneous and organized into sub-compartments that can release Ca^2+^ independently (Golovina and Blaustein, 1997, 2000). It would be fascinating to see if IP_3_R subtypes are located to specific functional domains, e.g., signaling domains of membrane receptors, ER-mitochondria contacts (Bartok et al., 2019), and ER-plasma membrane junctions (Thillaiappan et al., 2017). In addition to the ER, astrocytic IP_3_Rs can also be found on other Ca^2+^ stores, with unique properties, i.e., the large dense-core vesicles (Hur et al., 2010), which may be comparable to the thapsigargin insensitive, Bafilomycin A1 sensitive, acidic Ca^2+^ stores previously described in secretory cells (Gerasimenko et al., 1996, 2009, 2011; Hur et al., 2010). Notably, compared to the ER, acidic Ca^2+^ stores can exhibit enhanced sensitivity to IP_3_ (Yoo, 2010). Other potential IP_3_-sensitive Ca^2+^ stores include the nuclear envelope (Gerasimenko et al., 1995; Petersen et al., 1998), nucleoplasm (Echevarría et al., 2003), Golgi (Pinton et al., 1998), and plasma membrane (Dellis et al., 2006).

### Summary

While IP_3_R subtypes are regulated by IP_3_ and Ca^2+^ and have many common interacting partners, they differ in how they are affected by these regulators. Such differences enrich the diversity of spatio-temporal Ca^2+^ profiles created by IP_3_Rs. The IP_3_R subtype expression pattern, *in vivo*, is tissue specific and their subcellular localization is highly variable and dependent on cell types, and this carries important functional implications. Together with recent reports showing the distinct role of IP_3_R1 and IP_3_R3 in Bergmann glia and astrocytes, these facts support the view that IP_3_R isoforms 1 and 3 are unique Ca^2+^ channels that need to be addressed independently of IP_3_R2.

## Tools to Dissect the Role of Ip_3_R Isoforms

### Experimental and Analytical Tools

To understand the role of the various Ca^2+^ signals in astrocyte physiology, it will be necessary to make quantitative measurements (Neher, 2008). Progress in this direction has been frustrated by the unique astrocyte morphology and difficulties in interpreting recorded Ca^2+^-dependent fluorescent signals (Rusakov, 2015). To accurately capture Ca^2+^ dynamics in sub-cellular compartments, there is a need to adopt imaging techniques with improved resolution and to develop tools for efficient analysis in three-dimensional (Bindocci et al., 2017; Romanos et al., 2019). Because of our poor understanding of functional compartments, analysis of astrocytic Ca^2+^ dynamics has been moving toward state-of-the-art event-based analysis (Romanos et al., 2019; Wang et al., 2019; Bjørnstad et al., 2021), nevertheless ROI (region-of-interest) based analysis, informed by cellular anatomy (functional compartments) and molecular architecture, has been critical for understanding neuron physiology (e.g., spines and boutons). To this end, the identification of morphologically distinct subcellular compartments are promising targets for classical ROI based analysis, i.e., “glial microdomains” on Bergmann glial processes (Grosche et al., 1999) and “astrocytic compartments” on major branches (Panatier et al., 2011), both revealed using confocal microscopy, and astrocytic nodes and shafts within the spongiform structure visualized using live STED microscopy (Arizono et al., 2020). The ultimate goal of extracting quantitative Ca^2+^ dynamics from fluorescent data is non-trivial but achievable using realistic biophysical cell models (Rusakov, 2015; Denizot et al., 2019), a task simplified by the recent development of the open-source flexible model builder ASTRO (Savtchenko et al., 2018). For an accurate understanding of Ca^2+^ dynamics, it will be necessary to constrain models further using empirically determined details, e.g., receptor kinetics, expression patterns, endogenous Ca^2+^ buffering, etc.

### Pharmacological Tools

It is difficult to disentangle the physiological roles of IP_3_R subtypes in cells that typically express complex mixtures of homo- and hetero-tetrameric IP_3_Rs. There are no ligands that usefully distinguish among IP_3_R subtypes (Saleem et al., 2013a,b) and nor are there effective antagonists that lack serious side effects (Michelangeli et al., 1995). Of the available antagonists, heparin is currently the most useful. Heparin is a membrane impermeant pan-IP_3_R inhibitor that may be selectively loaded into astrocytes using a whole-cell patch-pipette (Sherwood et al., 2017). Recent developments report small impermeant competitive antagonists of IP_3_R1, which, compared to heparin, are likely to have fewer off-targets (Konieczny et al., 2016). Well-characterized function-blocking monoclonal antibodies are powerful tools to specifically inhibit IP_3_R subtypes (Miyazaki et al., 1992; Inoue et al., 1998; Nishiyama et al., 2000; Gerasimenko et al., 2009). This technology has not yet been applied to astrocytes.

### Genetic Tools

#### Inhibition of IP_3_ Induced Ca^2+^ Release

IP_3_R2KO and conditional-KO (cKO) mice (Petravicz et al., 2014; Padmashri et al., 2015; Wang et al., 2021) are widely used, however, as highlighted above, deletion of IP_3_R2 does not abolish IICR. IICR can be suppressed, irrespective of the underlying receptor, using an IP_3_-sponge to buffer IP_3_ (Xie et al., 2010; Tanaka et al., 2013), or an IP_3_-5′-phospatase transgene to enhance IP_3_ metabolism (Kanemaru et al., 2007; Foley et al., 2017). To study the physiological role of IP_3_R subtypes it is necessary to develop inducible cKO for IP_3_R1 (Sugawara et al., 2013) and IP_3_R3. Specific knock-down of IP_3_R subtypes can also be achieved using viruses to introduce short hairpin RNA into astrocytes (Shiratori-Hayashi et al., 2020).

#### Activation of IP_3_ Induced Ca^2+^ Release in Astrocytes

##### Pharmacogenetics

DREADDs (designer receptor exclusively activated by designer drug) enable the selective activation of GPCR-IP_3_R-Ca^2+^ signaling in astrocytes. The most used DREADDs are the excitatory Gq or inhibitory G_i_-coupled receptors, hM3Dq and hM4Di, respectively (derived from human M3/M4 muscarinic receptor). Both receptors are activated by a pharmacologically inert but bioavailable ligand clozapine-N-oxide (CNO) while being non-responsive to endogenous GPCR ligands (Agulhon et al., 2013). hM3Dq has been used to demonstrate an astrocytic role in behaviors such as food intake (Yang et al., 2015), fear response (Martin-Fernandez et al., 2017), and memory recall (Adamsky et al., 2018). While the DREADDs enables selective activation of astrocytes, they have two major drawbacks: Firstly, the exogenous receptors have not been targeted to specific signaling domains and are likely to be spatially uncoupled from signaling nanodomains critical to IP_3_R physiology (Bootman et al., 2001); Secondly, because of the sustained (hour-long) activation by exogenous ligands (Iwai et al., 2021), temporal features of astrocyte signaling are lost. While perhaps mimicking global Ca^2+^ surges, the available DREADDs are unlikely to recapitulate many of the local Ca^2+^ transients typically observed within fine astrocytic processes (Shigetomi et al., 2013a; Arizono et al., 2020).

##### Optogenetics

To achieve temporal control, an optogenetic approach has been developed for the reliable stimulation of endogenous GPCR-IP_3_R-Ca^2+^ signaling cascade using light. Light activation has been achieved by introducing to astrocytes either a mammalian light-sensitive G_q_/G_i/o_-protein-coupled photopigment, Melanopsin (Panda, 2005; Bailes and Lucas, 2013; Mederos et al., 2019), or light-activated chimeric GPCRs, termed OptoXRs. OptoXRs are generated by replacing the intracellular loops of a light-sensitive GPCR, e.g., rhodopsin, with those of a donor GPCR, e.g., G_q_-coupled human adrenergic receptor α_1a_ (Airan et al., 2009; Figueiredo et al., 2014; Tang et al., 2014; Adamsky et al., 2018; Iwai et al., 2021).

### Summary – Future Developments

While having great potential for controlling astrocytic activation, a central question is to what extent do the chimeras mimic the signaling of wild-type receptors. GPCRs can have multiple signaling axis, e.g., multiple G-protein axes, β-arrestins, wnt-frizzled, or the hedgehog-smoothened axes (Bailes and Lucas, 2013; Tichy et al., 2019), and the signaling axes bias is often not characterized but can have profound side effects on physiology (Agulhon et al., 2013; Tichy et al., 2019). Indeed, the functional outcome of activating G_q_ in astrocytes using different exogenous receptor (i.e., hM3Dq and MrgA1) is not reproducible (Agulhon et al., 2010; Adamsky et al., 2018). While multiple signaling axis could confuse the role of IICR, they may be required to obtain an optimal IP_3_R response (Konieczny et al., 2017). Another primary concern is that DREADDs and optoXRs likely activate IP_3_Rs from cellular compartments distinct from those of the endogenous receptors, limiting their ability to recreate physiologically relevant Ca^2+^ profiles. To address this, next-generation activation tools are being engineered to mimic the subcellular distribution of endogenous receptors (Oh et al., 2010; Masseck et al., 2014; Spoida et al., 2014; Tichy et al., 2019).

In the last decade, substantial progress has been made revealing diverse spatio-temporal Ca^2+^ signaling in astrocytes. Understanding the subtleties of these signals will require detailed knowledge of the astrocytic Ca^2+^ signaling toolbox along with the generation and characterization of more sophisticated tools to control and accurately recapitulate the physiologically relevant Ca^2+^ signals.

## Author Contributions

MS and MA performed the literature survey, wrote the manuscript, and prepared the figures and tables. MS, MA, AP, KM, and SHRO reviewed, finalized, and approved the final version.

## Conflict of Interest

The authors declare that the research was conducted in the absence of any commercial or financial relationships that could be construed as a potential conflict of interest.

## Publisher’s Note

All claims expressed in this article are solely those of the authors and do not necessarily represent those of their affiliated organizations, or those of the publisher, the editors and the reviewers. Any product that may be evaluated in this article, or claim that may be made by its manufacturer, is not guaranteed or endorsed by the publisher.
